# EGFR mutations cause a lethal syndrome of epithelial dysfunction with progeroid features

**DOI:** 10.1002/mgg3.156

**Published:** 2015-06-04

**Authors:** Rebecca Ganetzky, Erin Finn, Atrish Bagchi, Ornella Zollo, Laura Conlin, Matthew Deardorff, Margaret Harr, Michael A Simpson, John A McGrath, Elaine Zackai, Mark A Lemmon, Neal Sondheimer

**Affiliations:** 1 Department of Pediatrics, The University of PennsylvaniaPhiladelphia, 19104, Pennsylvania; 2 Division of Genetics, The Children’s Hospital of PhiladelphiaPhiladelphia, 19104, Pennsylvania; 3 Division of Biochemical Genetics, The Children’s Hospital of PhiladelphiaPhiladelphia, 19104, Pennsylvania; 4 Department of Biochemistry and Biophysics and Graduate Group in Biochemistry and Molecular Biophysics, The University of PennsylvaniaPhiladelphia, 19104, Pennsylvania; 5 Department of Pathology, The University of PennsylvaniaPhiladelphia, 19104, Pennsylvania; 6 Department of Medical and Molecular Genetics, King’s College LondonLondon, United Kingdom; 7 St. John’s Institute of Dermatology, King’s College LondonLondon, United Kingdom; 8 The Centre for Dermatology and Genetic Medicine, University of DundeeDundee, United Kingdom

**Keywords:** Epidermal growth factor, ichthyosis, progeria, receptor protein-tyrosine kinase

## Abstract

The epidermal growth factor receptor (EGFR) is part of a large family of receptors required for communicating extracellular signals through internal tyrosine kinases. Epidermal growth factor (EGF) signaling is required for tissue development, whereas constitutive activation of this signaling pathway is associated with oncogenic transformation. We identified homozygous c.1283G>A (p.Gly428Asp) mutations in the extracellular domain of *EGFR* in two siblings. The children were born prematurely, had abnormalities in skin and hair, suffered multisystem organ failure, and died in the neonatal period from intestinal perforation. EGF failed to induce mutated receptor phosphorylation in patient-derived fibroblasts and activation of downstream targets was suppressed. The heterologously expressed extracellular domain was impaired in stability and the binding of EGF. Cells from the affected patient undergo early senescence with accelerated expression of *β*-galactosidase and shortened telomeres at all passages when compared to controls. A comparison of homozygous inherited regions from a separate report of a patient from the same ethnic background and *EGFR* genotype confirms the pathogenicity of *EGFR* mutations in congenital disease.

## Introduction

Epidermal growth factor receptor (EGFR, OMIM 131550) plays critical roles in organismal development by transducing an extracellular signal to guide growth and differentiation of multiple tissue types (Sibilia et al. 2007). The disruption of *EGFR* in mouse model systems causes strain-specific lethality and prominent effects upon heart, brain, and epithelial tissues (Sibilia and Wagner 1995; Threadgill et al. 1995; Hansen et al. 1997). Naturally occurring mouse models with point mutations in *EGFR* (*waved-2*) have defects in hair and eyelid opening (Luetteke et al. 1994).

Studies of the role of EGFR in organ and tissue development have been nearly overshadowed by the discovery of the role of activating *EGFR* mutations in tumor biology (Pao and Chmielecki 2010). *EGFR* is overexpressed in a range of epithelial tumor types, and somatic mutations in *EGFR* were found to mediate the susceptibility of non-small cell lung cancer to gefitinib, a chemotherapeutic that inhibits the kinase activity of *EGFR* (Lynch et al. 2004). Although several agents have been developed to block EGFR activity, resistance through the activation of alternative kinases remains a ubiquitous problem (Niederst and Engelman 2013).

Investigators recently described a homozygous mutation in the extracellular domain of *EGFR* (c.1283G>A (p.Gly428Asp)) in a patient with a profound inflammatory skin disease who died from cutaneous infection (Campbell et al. 2014). These features mimicked dermatological complications that are typically seen in patients treated with agents that inhibit EGFR activity (Agero et al. 2006). We report here two siblings with the identical mutation with a related phenotype causing death in the neonatal period from intestinal perforation. Our studies confirm that loss of function mutations in *EGFR* causes a complex syndrome with both progeroid phenotypic and cellular features.

## Material and Methods

### Molecular confirmation

Amplification and sequencing of exon 11 of *EGFR* was performed using: 5′ AGCCTCTTCGGGGTAATCAG and 5′ TGCTTCTGTGTCCACTCCAG.

### Downstream targets

Dermal fibroblasts from patient 2 and an unrelated control were incubated with 50 ng/mL epidermal growth factor (EGF) for 15 min. The expression of *JUN*, *MYC*, and *FOS* was analyzed on 2 *μ*g of cDNA for each sample by real-time PCR (Applied Biosystems, Grand Island, NY, USA). Three experiments were performed on four replicates of each sample. Representative data from one experiment are shown. *GAPDH* expression was used as a control. Statistical significance was determined using one-way analysis of variance.

### Progeroid analysis

Telomere length was quantitated using TeloTAGGG Telomere Length Assay (Roche, Basel, Switzerland) according to the written protocol. *β*-galactosidase staining was performed using the Senescence *β*-galactosidase kit (Cell Signaling, Danvers, MA, USA).

### Serial passaging

Dermal fibroblasts from patient 2 and a control individual were grown in Dulbecco’s Modified Eagle Medium (DMEM) (Life Technologies, Grand Island, NY, USA), supplemented with 10% fetal bovine serum (FBS) and 0.5% uridine. Cells were plated in triplicate at a cellular density of 100,000 cells/well weekly from sequential passages. Cells were counted every week from passage 6 to 23.

### Western blots

Nearly confluent fibroblasts were serum starved for 24 h. Cells were incubated with EGF at a concentration of 50 ng/mL for 0, 30, or 60 min. Immunoblotting was performed using EGFR antibodies at a concentration of 1:2000 (Thermo scientific, Danvers, MA, USA), and phospho-EGFR antibodies at a concentration of 1:1000 (Cell Signaling, Danvers, MA, USA).

### Protein purification

Histidine-tagged sEGFR^WT^ and sEGFR^G428D^ were expressed in baculovirus-infected Sf9 cells as previously described (Ferguson et al. 2000; PMID 10970856) and purified by nickel-NTA and size exclusion chromatography. Protein was >80% pure by Coomassie staining.

### Surface plasmon resonance

EGF was immobilized by amine coupling to an activated CM5 surface as previously described (Ferguson et al. 2000; PMID 10970856). sEGFR^WT^ and sEGFR^G428D^ proteins were flowed over this surface as well as a control surface at a flow rate of 10 *μ*L/min for 10 min, which was sufficient to reach equilibrium even at low receptor concentrations. The relative responses shown in Figure5 were fit to Langmuir single-site binding isotherms in Prism 6.0 (Graphpad, La Jolla, CA, USA).

### Growth curve

Control and patient fibroblasts were plated at a cellular density of 50,000 cells/well. Three media conditions were used: DMEM supplemented with 10% FBS, or DMEM supplemented with 5 *μ*g/mL insulin, 40 ng/mL dexamethasone, and 50 *μ*g/mL ascorbic acid with or without 50 ng/mL of EGF.

### Variant submission

This variant has been submitted to the Leiden Online Variant Database, a public gene variant database. This information can be accessed at http://egfr.lovd.nl. The reference wild-type sequence is RefSeq NM_005228.3.

## Results

### Patient phenotype

**Figure 1:**
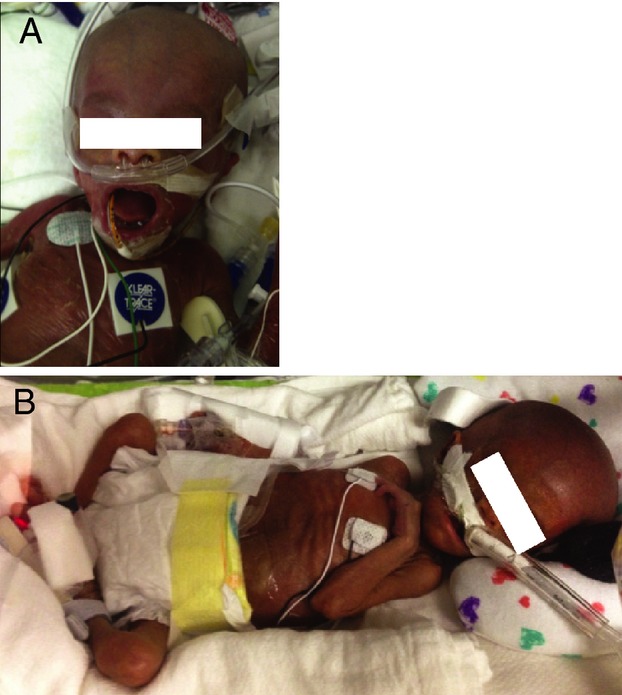
Physical features of the affected siblings. The sister (A) was photographed at 2 months of age and the brother (B) at 3 weeks of age.

We evaluated two siblings from a consanguineous Roma family. *Patient 1* was born at 34 weeks gestation. She had severe intrauterine growth restriction, nephromegaly, and renal tubulopathy. Weight, length, and head circumference were less than the first percentile (at 2 months weight and head circumference were 50th percentile for 33.5 weeks’ gestation; length was 50th percentile for 32 weeks’ gestation). She had craniofacial dysmorphism with pseudohydrocephalus, a progeroid appearance, striking desquamation with ichthyotic, hyperpigmented, translucent skin, absence of subcutaneous fat and sparse, stiff hair (Fig.1A). She had progressive abdominal distention and passed away after intestinal perforation at 3 months of age.

*Patient 2* was born at 33 weeks gestational age following a pregnancy complicated by polyhydramnios requiring therapeutic amniocentesis. He had ventilator-dependent respiratory failure, grade IV intraventricular hemorrhage, pancytopenia, liver failure, and severe intrauterine growth retardation. His birth weight was 1195 g, and which is less than the first percentile, as were his length and head circumference (weight was 50th percentile for 29 weeks; length was 50th percentile for 30.5 weeks and head circumference was 50th percentile for 31 weeks.) He had a similar appearance to patient 1 (Fig.1B) with apparent macrocephaly and an inverted triangular appearance to his face, a progeroid appearance; thin, translucent hyperpigmented, dry and ichthyotic skin, absence of subcutaneous fat, absent scalp hair, and sparse eyebrows. A skin biopsy was performed and showed thin dermis, lymphocytic infiltration of hair follicles, and wiry appearing collagens (Table1).

**Table 1:** Comparison of phenotype between the patients reported here, the patient reported by Campbell et al. (2014) and the waved-2 mouse model

	Patient 1	Patient 2	Campbell et al.	Waved-2 mouse model
Polyhydramnios	−	+	+	−
Premature birth	+ (34 weeks)	+ (33 weeks)	+ (34 weeks)	−
IUGR	+	+	+	+
Alopecia	+	+	+	+
Aged facial appearance	+	+	−	N.A.
Pseudohydrocephalus	+	+	−	−
Skin desquamation	+	+	+	Abnormal skin architecture
Ichthyosis	+	+	+	N.A.
Acquired skin inflammation	N.A.	N.A.	+	+
Absent subcutaneous fat	+	+	−	−
Trichomegaly	−	−	+	N.A.
Nephromegaly	+	+	+	Renal malformations
Intestinal perforation	+	+	−	Susceptible to colonic injury
Recurrent vomiting/diarrhea	+	+	+	−
Recurrent infections	−	−	+	−
Respiratory difficulties	−	+	+	−

Genomic DNA from patients 1 and 2 was analyzed by single-nucleotide polymorphism microarrays. Two large shared regions of homozygosity were identified; a 2.8 Mb region on chromosome 2p (chr2:38,029,531-40,857,899) and a 32.9 Mb region on chromosome 7 chr7: 45,297,878-78,234,264.) Whole-exome sequencing identified four homozygous mutations between these two regions in patient 1, as well as two shared homozygous changes outside of the large regions of homozygosity (Table S1). *EGFR*:c.1283G>A (p.Gly428Asp) was selected for further analysis as the other mutations lacked apparent relevance to the phenotype.

### EGF signaling is impaired in patient cell lines

**Figure 2:**
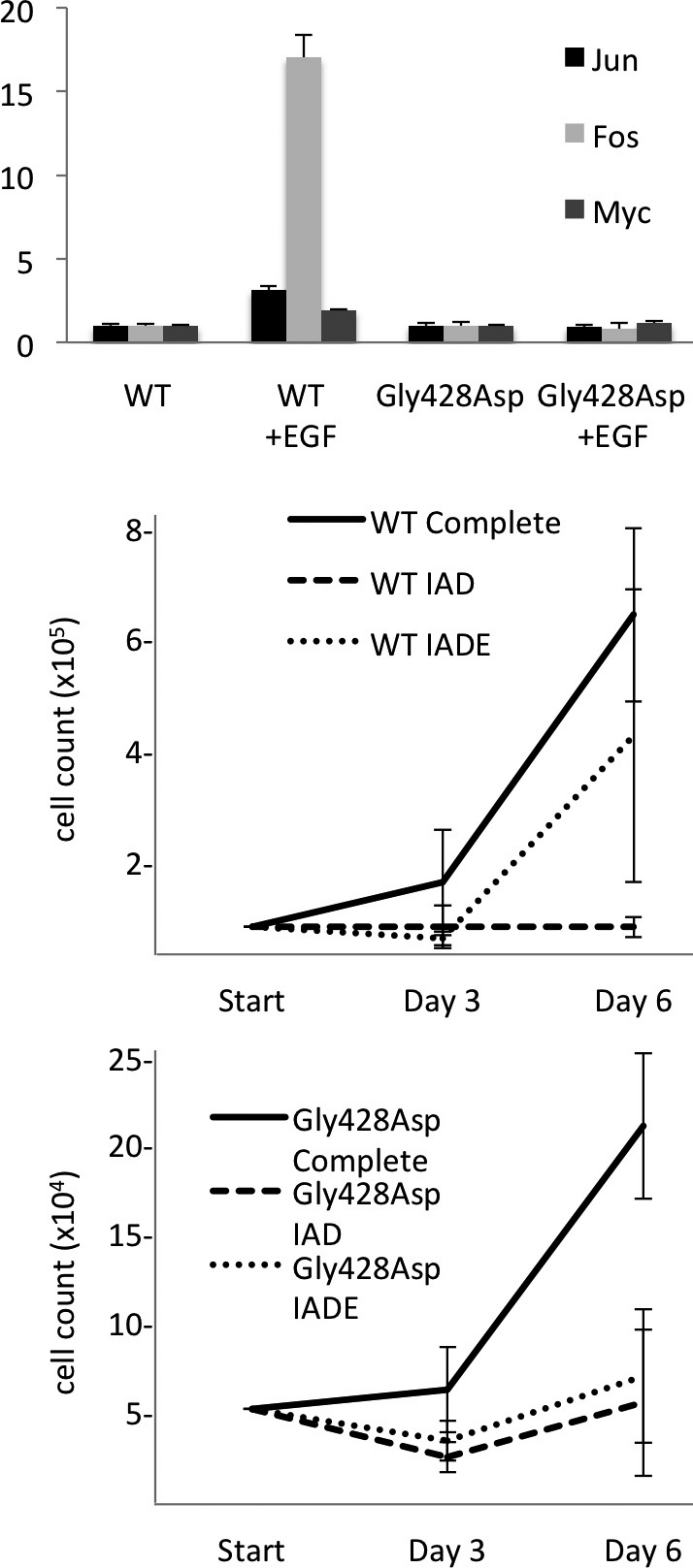
Downstream targets of EGF, Jun (dark bars), Myc (grey bars), and Fos (light bars), are increased in response to stimulation with EGF in control cells, but not patient cells (A). Growth of control cells in a minimal media of insulin, dexamethasone, and ascorbic acid (IAD) (lightest line) can be rescued with EGF treatment (IADE) (middle line) (B), whereas patient cell growth cannot be rescued with EGF (middle line) (C).

Binding of EGF by EGFR activates a sequence of receptor tyrosine kinases and regulates nuclear transcription. Dermal fibroblast cell lines were generated from patient 2 and a control individual. EGF-induced expression of *JUN*, *FOS*, and *MYC* were assessed using real-time PCR. Despite robustly inducing these immediate early gene products in control fibroblasts as expected (Fig.2A), EGF was not able to increase their expression in the patient-derived cells (*JUN, P* = 0.0001 *FOS*: *P* < 0.0001*, MYC*: *P* < 0.0001*;* Fig.2A). Similarly, whereas EGF supplementation will allow control fibroblasts to grow in a minimal medium containing insulin, ascorbic acid, and dexamethasone (IAD), EGF failed to support the growth of patient-derived cells in the same medium (Fig.2B and C) – reflecting a loss of *EGFR* function.

### Direct assessment of EGFR Function

**Figure 3:**
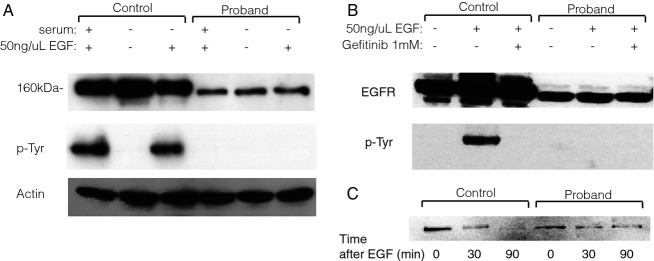
Western blot shows higher levels of EGFR in control compared to patient samples, and higher levels of p-EGFR following EGF stimulation in controls samples, as compared to patients (A). Addition of the EGFR kinase inhibitor gefitinib eliminates EGFR phosphorylation following EGF stimulation and does not change overall EGFR levels (B). Levels of EGFR are decreased at 30 and 90 min following EGF stimulation in control cells, but are unchanged at the same time points in patient cells (C).

To evaluate levels of EGFR expression and ligand-induced activation, we probed western blots of cell lysates with antibodies against EGFR and phospho-EGFR. EGFR levels in the patient-derived cell line were substantially reduced in comparison to an unaffected control (Fig.3A, top). Tyrosine autophosphorylation of EGFR in response to EGF or serum addition was robust in control cells. (Fig.3A, bottom). but could not be detected in patient-derived samples. This suggests that both the level and activity of EGFR^Gly428Asp^ are strongly impaired. To confirm that the observed phosphorylation in control cells was EGFR-specific, we repeated the experiment in the presence of the EGFR inhibitor gefitinib, which entirely inhibited phosphorylation in the control cells and lacked any effect in the patient sample (Fig.3B).

Previous studies have shown that EGFR levels decrease following activation as the receptor is internalized and degraded (Beguinot et al. 1984). We reasoned that if the EGFR: c.1283G>A (p.Gly428Asp) mutation prevented proper localization of EGFR or its ability to bind to EGF, we should see no alteration of receptor levels following administration of the ligand in the patient cells. Indeed, whereas EGF strongly reduced the levels of EGFR at 30 and 90 minutes following stimulation of wild-type cells, it had no detectable effect upon the EGFR^Gly428Asp^ levels in the patient cell line (Fig.3C). These results build upon the previous finding that EGFR^Gly428Asp^ is mislocalized to the cytoplasm, where it is unaffected by ligand (Campbell et al. 2014).

### EGFR^Gly428Asp^ binds to EGF with reduced affinity in vitro

**Figure 4:**
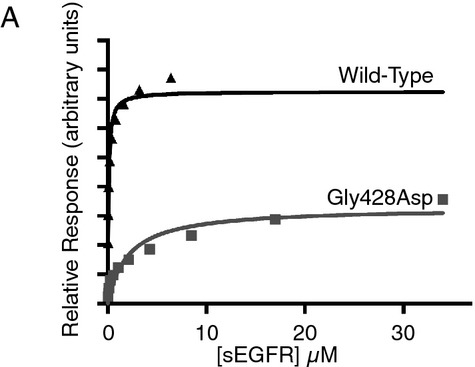
Binding of wild-type and G428D sEGFR to immobilized EGF, assessed using surface plasmon resonance (SPR). Equilibrium responses relative to a blank surface are plotted as a function of injected receptor concentration.

To evaluate the effect of the EGFR:c.1283G>A (p.Gly428Asp) mutation on the ability of EGFR to bind its ligands, we generated purified recombinant EGFR extracellular region (sEGFR) as described and used surface plasmon resonance to measure ligand binding as described (Ferguson et al. 2000). During purification of the c.1283G>A (p.Gly428Asp)-mutated sEGFR variant (sEGFR^Gly428Asp^), it was found to run as a broad peak in size exclusion chromatography, indicating a degree of misfolding and aggregation. The resulting purified sEGFR^G428D^ protein bound EGF approximately 100-fold more weakly than wild-type sEGFR (Fig.4). These data suggest that the *EGFR*:c.1283G>A (p.Gly428Asp) mutation causes EGFR’s extracellular region to fold incorrectly, consistent with both reduced levels of the protein in patient-derived cells (and efforts to express *EGFR*^Gly428Asp^ exogenously) and loss of EGF binding. Glycine 428 is on one of the ligand-binding domains in EGFR (domain III), but is distant from the ligand-binding site. Its substitution with an aspartate would project that acidic residue into the hydrophobic core of domain III, likely resulting in impaired folding and/or stability.

### Analysis of cellular senescence

**Figure 5:**
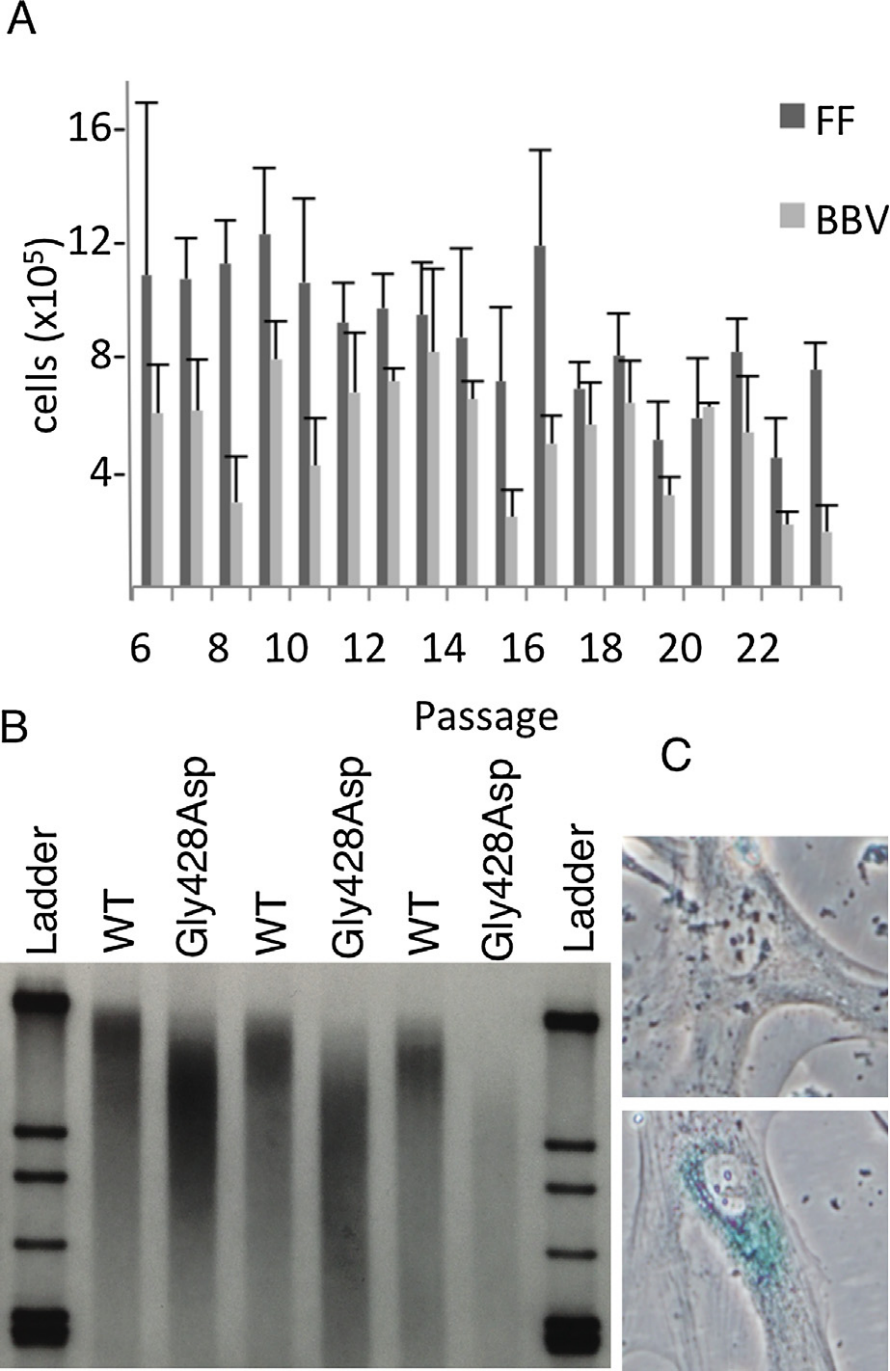
Increased senescence is associated with the G428D mutation as evidenced by decreased replicative capacity over serial passages (A), increased telomere shortening over serial passages (B), and increased beta-galactosidase staining (C).

Because of the progeroid appearance of the patients, we evaluated several markers of cellular senescence. Telomere length was shorter in patient cells as compared to controls and the observed shortening of telomere length in culture was accelerated (Fig.5A). We evaluated the replicative capacity of patient and control fibroblasts by serially passaging cells and found that the *EGFR*^Gly428Asp^ patient cell line had impaired replication at late passage when compared to control cells (Fig.5B). Finally, we examined senescence-associated beta-galactosidase staining. At passages where there was no notable staining in control fibroblast cell lines we found extensive beta-galactosidase expression in patient cells (Fig.5C).

### Genomic comparison to previously reported patient

The identification of a previously undescribed mutation in patients from the same ethnic background in this study and that of Campbell, et al. suggests that the patients may be related; however, there is no overlap in the pedigrees reported by the families, and the patient seen by Campbell et al. (2014) was seen in Poland and the United Kingdom, while the patients reported here have lived in Eastern USA for multiple generations. Therefore, to further assess relatedness, we compared exome data from our patient 1 and the previously reported patient (Fig. S1) We selected 75 very rare variants (<1% of the general population) reported in our patient. We found that six of those rare variants were shared between the two patients, spread across several chromosomes. *EGFR*^Gly428Asp^ was the only rare homozygous variant shared between the two patients. Supporting the hypothesis of a shared ancestor, both patient 2 and the patient reported by Campbell et al. (2014) share a polymorphism in *EGFR*, which is present in 30% of the general population. However, the shared chromosomal region on chromosome 7 is maximally 600 KB based on the most proximal unshared mutations. We conclude that the patients are likely related but the common ancestors are distant.

## Discussion

Receptor tyrosine kinases are used to mediate a broad variety of extracellular to nuclear signals for growth and differentiation. Defects in several of these proteins cause recognizable syndromes including defects in the insulin receptor in Donohue syndrome and *NTRK1* in Type II familial dysautonomia (Robertson et al. 2000). Evidence in this study and in the analysis of an additional affected patient from the same ethnic background shows that a failure of EGFR signaling leads to a complex phenotype with marked defects in epithelial formation and gut integrity.

Based upon our evidence, we suggest that EGFR^Gly428Asp^ has a range of defects. First, the protein has reduced stability and binding of EGF in vitro. Second, EGF cannot stimulate receptor tyrosine kinase activity. Finally, the reduced levels of EGFR^Gly428Asp^ in unstimulated cells suggest that the protein is either unstable or localizes to a cellular compartment where its turnover is increased.

These findings are consistent with the previous findings by Campbell, et al. Both this study and the previous study found a reduced quantity of EGFR and that the protein present is poorly stimulated, which is consistent with either a defect in the EGF-binding domain, or cytoplasmic mislocalization of mutant EGFR as found by Campbell, et al. The previous study showed an increased amount of constitutive endocytosis, which helps explain the low levels of EGFR. Here, we show that ligand-stimulated endocytosis, a physiologic process that initiates the EGFR signaling cascade, is decreased, which helps explain the decreased activation of downstream targets.

It appears that the patients in our study and that of Campbell, et al. are distantly related; however, the small size of the shared region, the limited shared genetic material, and the recurrence of disease in geographically distinct members of the same ethnic group is a concerning finding, suggesting that this mutation may be an underrecognized cause of congenital illness in the Roma population.

During our evaluation, we considered that the patients might phenotypically resemble other patients with Wiedemann-Rautenstrauch syndrome (WRS; also known as neonatal progeria), particularly in consideration of the facial shape, lack of hair, hyperpigmentation, and translucent skin. We evaluated samples from several other patients carrying this diagnosis and did not identify mutations in *EGFR* (data not shown). WRS remains a clinical diagnosis that lacks a clear etiology and may arise from several different genetic defects. It is possible that further studies may show that a subset of patients with WRS have mutations in *EGFR* and we would recommend screening suspected patients with WRS for *EGFR* mutations.

Amplification or activating mutations in *EGFR* has been observed in a wide range of human malignancies. Tumors with these genetic alterations are typically responsive to targeted therapies against EGFR, but invariably develop resistance to anti-EGFR therapies after exposure by the activation of alternative receptor tyrosine kinases. It is interesting to consider how developmental pathways in affected patients may have compensated for the loss of EGFR activity through the action of alternative receptor tyrosine kinase. Future studies of cellular development in the absence of EGFR signaling may provide important clues to the pathways that are used in the escape from EGFR inhibition.

## Supporting Information

**Figure S1.** Evaluation of 75 rare variants present in patient 1 in the patient reported by Campbell et al. shows multiple shared variants; only one rare variant is homozygous in both patients - *EGFR* G428D (A). Comparison of phenotype between our patients and the patient reported by Campbell et al. (B).

**Figure S2.** Pedigree of the affected patients shows an estimated coefficient of relationship of 6.25%. There is no known relationship between these patients and the patient reported by Campbell et al.

**Table S1.** Shared homozygous changes between patient 1 and patient 2. Only *EGFR* was selected as a putative candidate based on known gene function.
