# Supplementary material for: EGFR mutations cause a lethal syndrome of epithelial dysfunction with progeroid features
Source: Mol Genet Genomic Med. 2015 Jun 4;3(5):452–8. doi: 10.1002/mgg3.156 (PMC4585453; doi:10.1002/mgg3.156)
Supplement: Supplementary file 3 [file mgg30003-0452-sd3.docx]

| Gene Name | Mutation | RefSeq Accession | Genomic Position | Gene Function |
| --- | --- | --- | --- | --- |
| C7orf72 | c.782A>T (p.Glu261Val) | NM_001161834 | chr7:50135682-50198852 | Uncharacterized protein. |
| EGFR | c.1283G>A (p.Gly428Asp) | NM_005228.3 | chr7:55248979-55259567 | Epidermal Growth factor receptor. Receptor tyrosine kinase. EGFR knockout mice have absent hair follicles, complex skin abnormalities (hypoplastic) and succumb rapidly to systemic disease. |
| NSUN5P2 | c.575G>A  (p.Arg192His) | uc003twq.2 | chr7:72424050-72439870 | Noncoding RNA |
| FKBP6 | c.203C>T  (p.Met68Thr) | NM_003602 | chr7:72742155-72772641 | In William’s syndrome critical region. Animal model is aspermic. |
| HIGD1B | c.19T>C  (p.Trp7Arg) | NM_016438 | chr17:42923721-42927848 | Function not known. Linked to development and progression of pituitary adenomas |
| SIGLEC7 | c.100G>A  (p.Val34Met) | NM_014385 | chr19:51645558-51656783 | Mediates sialic-acid dependent binding to cells. May have inhibitory role on immune system activation; may also have a role in bone marrow differentiation. |

Table S1. Shared homozygous changes between patient 1 and patient 2. Only EGFR was selected as a putative candidate based on known gene function.
